# Antibiotic Use in Periodontal Therapy among French Dentists and Factors Which Influence Prescribing Practices

**DOI:** 10.3390/antibiotics10030303

**Published:** 2021-03-15

**Authors:** Kevimy Agossa, Kadiatou Sy, Théo Mainville, Marjolaine Gosset, Sylvie Jeanne, Brigitte Grosgogeat, Florence Siepmann, Florence Loingeville, Marie Dubar

**Affiliations:** 1 University of Lille, Inserm, CHU Lille, U1008—Controlled Drug Delivery Systems and Biomaterials, F-59000 Lille, France; kadia2sy@gmail.com (K.S.); florence.siepmann@univ-lille.fr (F.S.); 2 Department of Periodontology, School of Dentistry, University of Lille, Place de Verdun, 59000 Lille, France; theomainville@hotmail.fr (T.M.); marie.dubar@univ-lille.fr (M.D.); 3 Faculté d’Odontologie, Université de Lyon, Université Claude Bernard Lyon 1, 69008 Lyon, France; brigitte.grosgogeat@univ-lyon1.fr; 4 Hospices Civils de Lyon, Service d’Odontologie, 69007 Lyon, France; 5 Laboratory Orofacial Pathologies, Imaging and Biotherapies URP2496, Université de Paris, F-92120 Montrouge, France; drmarjolainegosset@gmail.com; 6 Service de Médecine Bucco-Dentaire, AP-HP, Hôpital Charles Foix, F-94200 Ivry-sur-Seine, France; 7 SFPIO—French Society of Periodontology and Oral Implantology, 44000 Nantes, France; 8 UFR d’Odontologie de Rennes, Bâtiment 15, 2 Avenue du Professeur Léon Bernard, Campus Santé, 35043 Rennes, France; doyen-odonto@univ-rennes1.fr; 9 Pôle Odontologie, CHU Rennes, 2 Rue Henri Le Guilloux, 35000 Rennes, France; 10 CNEP—French College of Teachers in Periodontology, 35000 Rennes, France; 11 Laboratoire des Multimatériaux et Interfaces, Université de Lyon—Claude Bernard Lyon 1, UMR CNRS 5615, F-69622 Villeurbanne, France; 12 ReCOL—French Private Dental Practice-Based Research Network, 69007 Lyon, France; 13 University of Lille, CHU Lille, ULR 2694—METRICS: Evaluation of Health Technologies and Medical Practices, F-59000 Lille, France; florence.loingeville@univ-lille.fr; 14 University of Lille, Inserm, CHU Lille, UMR-S 1172 JPArc, F-59000 Lille, France

**Keywords:** antibiotics, drug prescription, antibiotic stewardship, periodontal disease, survey

## Abstract

The aim of the present survey is to investigate the use of antibiotics during periodontal therapy among French dentists with a focus on exploring potential differences between various groups of practitioners. A self-administered questionnaire was distributed to different groups of practitioners including members of (i) the French Society of Periodontology and Implantology; (ii) the College of University Teachers in Periodontology and, (iii) private practitioners participating in the French general dental practice-based research network. 272 questionnaires were included in the analysis. Prescription patterns were globally in line with the current recommendations. Systemic antibiotics are most frequently used as a first-line therapy in necrotizing periodontitis (92%) and aggressive periodontitis (53.3% to 66.1%). However, malpractice still exists, including in the management of periodontal abscesses. Antibiotics are prescribed (i) less frequently for periodontal abscesses and (ii) more frequently for generalized aggressive periodontitis by members of the periodontal society and University college (*p* < 0.05). Amoxicillin (59.9%) and the amoxicillin + metronidazole (59.6%) combination were the most frequently prescribed molecules. Providing a high number of periodontal treatments per week, being more recently graduated, having a post-graduate certificate in periodontology and holding or having held an academic position/hospital practice were all factors associated with a better knowledge of and/or more adequate antibiotic use.

## 1. Introduction

Antibiotics have dramatically changed the way infectious diseases are treated. However their misuse and overuse contributes to the threat of antimicrobial resistance, which is a significant cause of morbidity and mortality worldwide [1,2,3]. France is among the countries where antibiotic consumption is the highest in the world, 30% above the European mean, especially in outpatient settings, which accounts for more than 90% of global antibiotic use in human medicine [4,5,6]. A large information campaign (“Antibiotic are not Automatic”) led to a significant reduction in the consumption of antibiotics in the community (up to 34%) between 2001 and 2005, but this positive effect was temporary. After a plateau phase between 2005 and 2010 the antibiotic consumption increased again from 2010 to 2018 (8%) [7]. The impact on the global burden of antimicrobial resistance in France varies according to the specific type of microorganism. Among Gram-positive bacteria, a significant reduction (−58%) in the proportion of MRSA (methicillin-resistant staphylococcus aureus) was observed between 2000 and 2016. However, the situation remains of concern for Gram-negative bacteria. The main issue is the increase in the resistance of *Enterobacteriaceae* to third-generation cephalosporins, the level of which has reached in 2017 12% for *Escherichia coli* and 35% for *Klebsiella pneumoniae* [7,8,9]. The preservation of antibiotic efficacy is part of the national Health strategy 2018-2022 and the prevention of antibiotic resistance has been one of the five priority topics of the French Sanitary Service for Health Students since 2021 [10,11].

It is clear that all health professionals (general practitioners, specialists, dentists, community pharmacists and nurses/allied healthcare professionals) have a role to play in primary prevention, patient education and control of the antimicrobial resistance burden but most research findings are related to general-practitioners (GPs) or inpatient settings [12,13,14]. Dental professionals should be fully engaged in antibiotic stewardship (AS) initiatives as dentists prescribe a significant proportion (8% to 11%) of antibiotics in outpatient settings [15]. Encouraging data from the NHS in the UK suggests that dentists responded well to AS and have reduced their antibiotic prescriptions more than in other fields of primary care between 2010 and 2017 [16]. While a number of studies have described dentists’ antibiotic prescribing patterns [17,18,19,20,21,22,23,24,25,26,27,28,29,30], a recent comprehensive review has pointed out the lack of data on the disease-specific use of antibiotics [31]. This information would be valuable in targeting appropriate areas of dental practice in antibiotic stewardship efforts.

Periodontitis is a frequent bacteria-induced inflammatory disease of the tooth supporting tissues which can result in tooth loss and may also affect patients’ general health and quality of life [32,33]. Severe periodontitis affects nearly 750 million people worldwide and is the 6th most frequent human disease [34]. The use of antibiotics in periodontal treatment is based on the infectious nature of the disease and their additional short- to mid-term clinical benefits, attributed to their ability to eliminate pathogenic bacteria which are inaccessible to standard mechanical treatment [35,36,37,38,39,40,41,42,43,44,45]. However, a recent Cochrane systematic review found very little evidence of the effectiveness of long-term follow-up of antibiotics in periodontal therapy and no certainty for a minimally significant clinical benefit [46].

In view of these uncertainties and the risk of adverse effects, including the potential development of multidrug resistance (MDR), it is surprising that very few reports have examined antimicrobial use in periodontal therapy [47,48,49]. The aims of this study are (i) to describe the use of antibiotics in periodontal therapy among French dentists; (ii) to explore the differences between various practice-based groups of dentists; and (iii) to investigate individual factors that might affect antibiotic prescribing during periodontal therapy.

## 2. Results

### 2.1. Characteristics of Respondents

Total of 563 dentists participated in the survey, of which 513 participants logged onto the online questionnaire. About 272 questionnaires were completed sufficiently to be included in the analysis. 155 (56.92%) participants were classified as “specialized/orientated practice” (SOP) including 107 (39.33%) from the French society of periodontology and oral implantology (SFPIO), 12 (4.41%) from the College of University Teachers of Periodontology (CNEP), and 36 (13.23%) affiliated to both. Total of 117 (43.01%) participants were classified as “general dental practitioners” (GDP) including 84 (30.88%) from the French private dental practice-based research network (ReCOL) and 33 (12.13%) who declared no affiliation to any of the listed groups. The average completion time of the online questionnaire was less than 10 min. Table 1 shows the demographic profile, educational background, and practice characteristics of the respondents. Most are self-employed (82.3%) and graduated in France (88.6%). About 40.1% have a postgraduate certificate in periodontology. Nearly all of them reported providing non-surgical periodontal treatment (96.7%) and 65% reported providing surgical periodontal treatment. 34.9% receive less than 5 patients per week for periodontal therapy and 36.4% receive more than 10.

### 2.2. Knowledge and Use of Systemic Antibiotics

**Figure 1:**
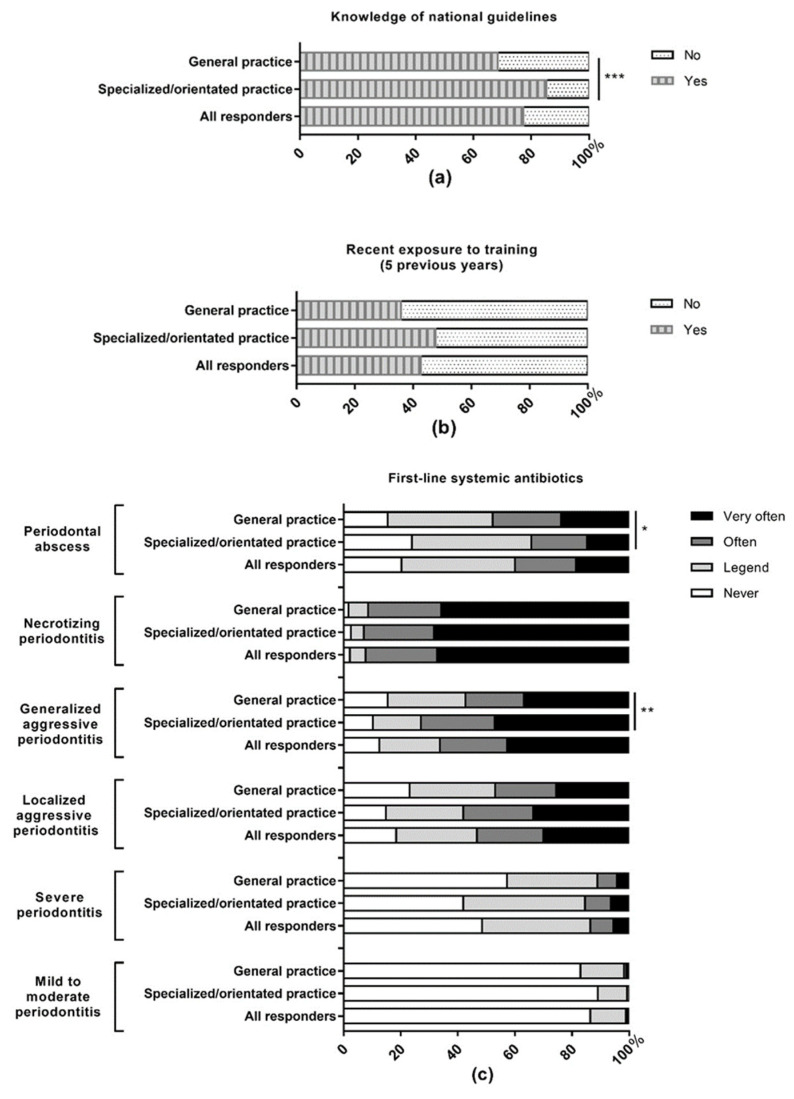

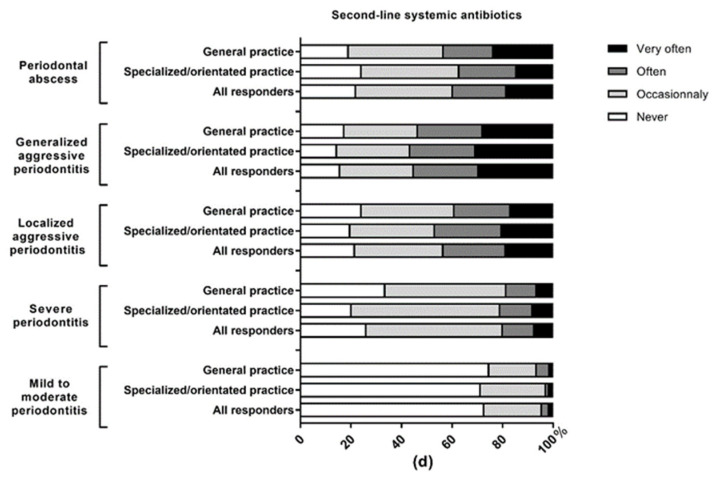
Use of systemic antibiotics in periodontal conditions by French dentists. (**a**) Knowledge of national guidelines about systemic antibiotics prescription; (**b**) exposure to training on systemic antimicrobial treatment in the previous 5 years; (**c**) use of antibiotics as a first-line periodontal therapy; (**d**) use of antibiotics as a second-line periodontal therapy. The data are presented as percentage and according to the affiliation of the practitioners. Specialized/orientated practice means that the dentist is affiliated to the SFPIO (French society of periodontology and oral implantology) and/or the CNEP (French college of University teachers in periodontology); general dental practice means that the practitioner did not declare membership in the SFPIO or CNEP. This include members of ReCOL (French general dental practice-based research network) and practitioners with no affiliation disclosed; * *p* < 0.05, ** *p* < 0.01, and *** *p* < 0.001.

Approximately three quarters (77.2%) of respondents declared knowing the national guidelines related to the use of systemic antibiotics (85.2% of periodontal society/college members vs 68.4% of general practitioners, *p* < 0.001) and 42.7% had been exposed to training on systemic antimicrobial treatment in the previous 5 years. Figure 1 presents the frequencies of antibiotic use in different periodontal conditions. Systemic antibiotics are most frequently used (“often” to “very often”) as a first-line therapy in necrotizing periodontitis (92% of all respondents); generalized aggressive periodontitis (66.1% of all respondents) and localized aggressive periodontitis (53.3% of all respondents). 40% of all dentists reported using antibiotics (“often” to “very often”) as a first-line therapy for periodontal abscesses compared to 13.6% in cases of severe periodontitis. As second-line therapy, antibiotics are the most frequently used (“often” to “very often”) in generalized (44.4%), and localized (43.7%) forms of aggressive periodontitis and periodontal abscesses (40%). Periodontal society/College members prescribe antibiotics more frequently (“often” to “very often”) as first line therapy in case of generalized aggressive periodontitis (72.9%, *p* < 0.01) and less frequently for periodontal abscesses (34.2%, *p* < 0.05) than general practitioners (57.3% and 47.8% respectively).

**Figure 2:**
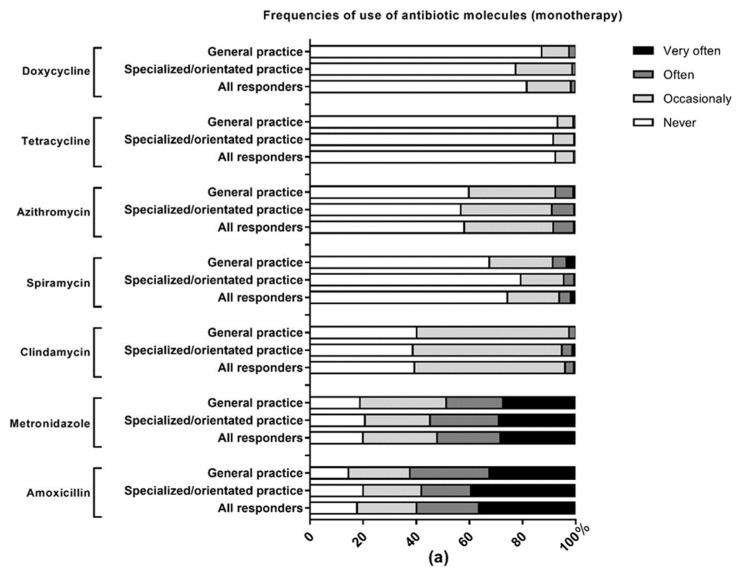

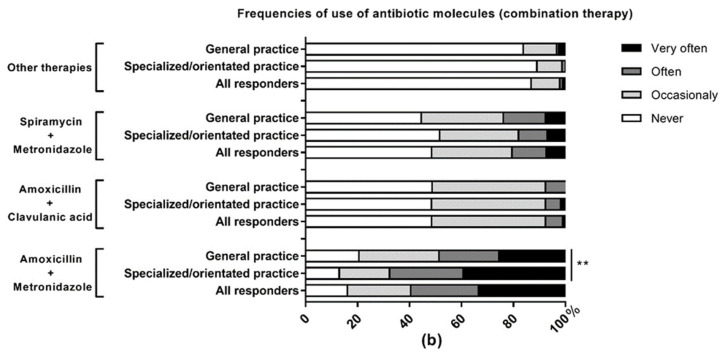
Use of antibiotic molecules in periodontal therapy. (**a**) Antibiotic molecules most frequently prescribed as monotherapy; (**b**) antibiotic molecules most frequently prescribed as combination therapy. The data are presented as percentage and according to the affiliation of the respondents. Specialized/orientated practice means that the dentist is affiliated to the SFPIO (French society of periodontology and oral implantology) and/or the CNEP (French college of University teachers in periodontology); general dental practice means that the practitioner did not declare membership in the SFPIO or CNEP. This include members of ReCOL (French general dental practice-based research network) and practitioners with no affiliation disclosed; ** *p* < 0.01.

Figure 2 presents the frequencies of use of the available antibiotic molecules in periodontal therapy. Amoxicillin (59.9%) and the combination amoxicillin + metronidazole (59.6%) are the most frequently prescribed molecules (“often” to “very often”) while tetracyclines (0.7%) and doxycycline (1.8%) are the least frequently used. Periodontal society/University College members prescribe more frequently (“often” to “very often”) the combination amoxicillin + metronidazole (67.7%) than general practitioners (48.7%) *p* < 0.01.

### 2.3. Knowledge and Use of Local Antibiotics

Half (50.4%) of the respondents declared knowing the national guidelines related to the use of local antibiotics (58.1% of periodontal society/college members vs 40.2% of general practitioners, *p* < 0.01) and 24.3% had been trained in local antimicrobial use through continuing education in the previous 5 years. 69.1% of participants knew of at least one local antimicrobial-delivery system but only 14.4% used these as a first-line therapy (11.8% occasionally) and 25% as a second-line therapy (22.4% occasionally) (Table 2). Higher usage of local antibiotics (minocyclin [parocline^®^]) was found in dentists having an orientated/specialized practice (Periodontal society/University College members) compared to the general dental practitioners (27.1% versus 15.4%, *p* < 0.05).

Among users, local antimicrobial use is more frequent (“often” to “very often”) in aggressive periodontitis (15.4% in localized forms and 11.6% in generalized forms) but no significant difference was found between specialized/orientated practice and general practice (Table 3). Among the non-users, almost half (49.4%) cited a lack of scientific evidence and 40.9% reported lack of experience as the reason (53.8% of general dental practitioners and 30.3% of specialized/orientated practitioners, *p* < 0.001) (Table 2).

### 2.4. Factors Influencing Knowledge and Practice Scores

The results of the multivariate analysis are presented in Table 4 and Table 5. The *p*-value of the global test of the regression model indicates that the fitted models were predictive at the 5% threshold.

The share of periodontics in the practice (*p* < 0.001), the year of graduation (*p* < 0.01) and postgraduate education, (*p* < 0.05) were all factors associated with an increased knowledge score. The higher the knowledge score, the more the practitioner is aware of the proper use of antibiotics. The year of graduation (*p* < 0.01) and postgraduate education (*p* < 0.05) were similarly associated with high practice scores and so was being or having been in an academic position (*p* < 0.001). The higher the practice score, the more prescribing patterns stated are consistent with the national guidelines. No correlation was found between knowledge and practice scores.

## 3. Discussion

### 3.1. Main Results and Comparison to Previous Studies

This study aimed to describe the self-reported practices of French dentists toward antibiotic use in periodontal therapy and assess the appropriateness of antibiotic prescription for this indication. It is worth mentioning that the periodontal pathogens, mostly anaerobes, are involved in various oral infection processes but can also disseminate through enteral and hematogenous route [50,51,52]. A recent study shows an increase of *E. faecalis* in the subgingival biofilm associated with periodontitis (9.8% in periodontitis, 7.8% in gingivitis and 2.2% in periodontal health *p* < 0.05) as well as high rates of low sensitivity/resistance (>64%) to at least one antimicrobial including antibiotics commonly used in dentistry [53]. This highlights the importance of dental antimicrobial stewardship.

Importantly, the present study focuses on exploring potential differences between various groups of practitioners in an effort to identify practice-based factors that could explain prescribing patterns. The objectives of the present study were different, although complementary to those of a national survey recently conducted in France [54]. This survey focused on the prophylactic use of antibiotics, oral surgery, and prescription in the context of dental emergencies. In this study, only 2% (*n* = 9) of the participants stated having an oriented or exclusive practice in periodontics. It should be kept in mind that there are no data on the number of practitioners practicing periodontics on an exclusive or dominant basis in France. In the present study, more than one-third of the participants declared receiving more than 10 patients per week for periodontal care, 40.1% hold a post-graduate certificate in periodontology and 65% perform periodontal surgeries. Interestingly, the post-graduate certificate and the number of periodontal treatments practiced per week were associated with a better knowledge and a more adequate use of antibiotics in periodontal therapy whereas membership in the SFPIO or CNEP was not. Considering the differences previously reported between specialists and general dentists, this result may indicate that in the study population, educational background and effective practice are more relevant for defining a dentist with an oriented/specialized practice than membership in a society or college [47,48,49]. In comparison, a recent national multicentric cross-sectional survey of knowledge, attitudes, and practices in Italy revealed no significant differences according to specialization or type of practice (hospital or primary care settings), but this study included young doctors (<35 years) from all medical fields [55].

Approximately half to three quarters of the respondents report knowing the national recommendations, which is consistent with the proportion (75.3%) observed in a previous survey in France [54]. According to the literature, knowledge of guidelines among dentists and dental students varies widely (from as low as 1.9% to as high as 100%) depending on the detail being examined [56]. On the other hand, the unclear risk of desirability bias further limits the confidence in self-reported knowledge. Overall, this study found positive trends in the use of systemic antibiotics for periodontal therapy among French dentists who mostly use these drugs in necrotizing periodontitis, rapidly evolving periodontitis called “aggressive periodontitis” and severe forms of “chronic periodontitis”. Systemic antibiotic prescription patterns are thus globally in line with the national recommendations and the protocols described in the international literature [57,58,59,60,61]. However, deficits and malpractice still exist among French dentists. This is the case of the unexpected frequent use (40%) of antibiotics as first-line treatment for periodontal abscesses for which mechanical debridement and antiseptics are recommended in the majority of patients [57,61]. However, it should be noted that specialized/orientated practitioners use antibiotics significantly less often in case of periodontal abscess than general practitioners. In contrast, specialists use antibiotics more often in cases of generalized aggressive periodontitis. A similar result has been reported in a previous study and is probably explained by a greater number of cases of severe periodontitis being referred to specialists [47].

Local antibiotics are used by a small proportion of French practitioners (14% to 25%). This rate is comparable to that observed in 2001 in England (14.8% to 30.8%) and much lower than in Germany in 2016 (60.9%) where the use of local antimicrobials increased by 6.2% in 10 years [47,48]. With regard to barriers to the use of local antimicrobials, it is interesting to note that these systems are perceived as being more effective than mechanical treatment alone by the majority of English dentists (81.6%) [47] while nearly half of French practitioners believe that the evidence of their benefit is insufficient [43]. This is potentially related to the fact that local antibiotics are “not recommended” for periodontal therapy in French guidelines [57]. Nonetheless, the decision of whether to adopt a treatment in clinical practice could be a more complex issue, particularly for therapies in constant evolution. Greenstein and Polson speculated that differences could exist between general practitioners and specialists [62]. While the specialist might assess the benefit of the treatment as modest based on the data from the literature, the general practitioner might find it useful, particularly if it is easy to use and limits the need for referral to a specialist [62]. In another study, lack of local postgraduate training would discourage the use of local antibiotics according to general dentists [47]. This is in line with our findings as significantly more general practitioners point to lack of experience as a barrier to local antimicrobial use while specialists point to lack of outcomes.

Postgraduate education, a more recent year of graduation, and treating a high number of periodontitis patients per week were factors associated with a higher level of knowledge and/or practice in antibiotic prescribing. Factors that may influence the awareness among dentists regarding antibiotic use have been discussed in many studies. Post-graduate training was repeatedly associated with an increased level of knowledge of the respondents, whereas increasing age and time spent in practice had a deleterious effect [56].

### 3.2. Limitations of the Present Study

The results of this survey must be interpreted in light of a number of limitations. In this study, a self-administered questionnaire was made available for participants through websites and professional social networks. Therefore, it is evident that (i) the results only reflect subjective estimates of respondents and, (ii) an exact response rate cannot be calculated since the number of people who actually received the invitation to participate in the study is unknown. However, it could be estimated that 15.47% (of the 924 active members of the SFPIO), 73.84% (of the 65 attendees to the national college of University teachers in periodontology in 2019), and 19.85% (of the 423 active members of the ReCOL) participated in this survey. This method of questionnaire distribution may also have led to a bias in the selection of respondents’ profiles (those who connect to the website). Importantly, nearly 30% of the participants reported no membership in the three listed groups. We cannot ascertain if this is an omission or if non-members accessed the survey through social networks. Another issue may concern the representativeness of the sample studied. Nearly one-third of the respondents are academics and/or work in a hospital, while only 1% of French dentists practice currently in a hospital [54]. It can therefore be speculated that the dentists most aware of the proper use of antibiotics are over-represented in this sample. In this case, the level of knowledge and practice is probably overestimated compared to the general population of French dentists. Despite these limitations, this survey achieved its goal of describing and comparing antibiotic use among different profiles of French dental practitioners.

### 3.3. Perspectives of the Present Study

As prescribers, dentists are an integral part of the antimicrobial stewardship puzzle and must be targeted by initiatives tailored to the specific context of their practice. Three approaches are proposed herein in this regard:

- Education: It is clear that no antimicrobial stewardship program can be successful without education. The lack of education on the prescription of antibiotics and the issue of antimicrobial resistance during undergraduate or medical specialty training has been previously emphasized in France and other countries [63,64,65,66,67]. The results of this survey confirm post-graduate education as a determining factor in the prescribing habits of dentists and suggest that practitioners, particularly those who have been in practice for a long time, should be made more aware of the need to improve their practices in the prescription of antibiotics for periodontal therapy. The guidelines on implementing antimicrobial stewardship programs suggest that the culture of antimicrobial stewardship should be integrated early in the pre-clinical and clinical curriculum before certain attitudes and prescribing habits are formed [68]. According to the literature, only 40% of medical students are familiar even with the term “antimicrobial stewardship” [69]. It has also been observed that slightly less than one-third of dentists change their prescribing habits after they first graduated from dental school [31]. Importantly, “patient influence” has been identified as the most frequent factor influencing the prescription of antibiotics in primary care settings including dental care [70]. This suggests that not only healthcare professionals but also the general public need to be educated about the significance of antibiotic resistance and the importance of reducing the use of antibiotics in dental care.
- Development/update of guidelines: The effectiveness of implementing guidelines on the rate of appropriate use of antimicrobials is well documented in the literature [71]. They have the advantage of being accessible to a wide audience including non-specialists in the considered field and allow standardization and streamlining of practices. The most recent French national recommendations about the use of antibiotics in dentistry were published in 2011 [57]. A recent Cochrane review identified a total of 10 systematic reviews on adjunctive use of systemic antimicrobials in periodontal therapy published since 2014 and more than 20 randomized control trials since 2011 [72]. Key changes have also recently been made to how periodontal diseases are diagnosed and classified. The implementation of a new classification scheme for periodontal and peri-implant diseases has resulted in a S3 Level Clinical Practice Guideline (CPG) proposed by the European Federation of Periodontology to facilitate the use of the most appropriate interventions, according to the stage and grade of the disease [61]. Therefore, updated national recommendations about the use of antibiotics in dentistry, which represent the current state of science, would be desirable to better inform practitioners in making their decisions. Fortunately, French dentists are favorable to receiving up-to-date training on antibiotic use. 43.7% report feeling inadequately informed and trained on this subject and 93.7% are willing to receive regular updates on prescribing recommendations, particularly in the form of practical sheets [54].
- Complementary approaches: used alone, didactic passive educational materials are insufficient as antimicrobial stewardship activities. They should be used in conjunction with complementary approaches such as prospective audit and continuous feedback, which have been demonstrated to decrease the number of new prescriptions and to improve clinician satisfaction [68,73]. For example, Computer-Assisted Decision Support Programs can provide real-time feedback that has been shown to result in significant reductions in the use of antimicrobials and an increase in concordance with recommendations [74,75,76].
- Implementation of practical public health actions: An operational strategy has been proposed in 2016 by the French Ministry of Health which includes 13 measures to control antibiotic resistance [77]. We fully adhere to this roadmap and we believe that the focus should be placed on the participation of all health professionals including dentists for whom few visible actions have been implemented so far. In terms of education, we propose (i) the implementation of a mandatory course on antimicrobial resistance for all medical and dental undergraduates or residents as well as (ii) a mandatory course at regular intervals for dentists already in practice. Similar measures already exist with regard to in-office radiation protection skills and training in emergency procedures and care [78]. In clinical practice, (iii) the setting-up of a network of sentinel dentists, similar to the existing network of sentinel medical doctors set up in 1984 [79] to ensure continuous monitoring of indicators of antibiotic consumption and antimicrobial resistance. With regard to research (iv) a support for innovation in the field of alternative antimicrobial strategies to antibiotics in dental practice and the development of research on prescribing practices in dentistry. Hopefully, the results of these studies will enable stakeholders to better understand the prescribing patterns of dentists and to better involve them in the collective fight against antimicrobial resistance.

## 4. Materials and Methods

### 4.1. Study Design

This study comprised a cross-sectional national survey on awareness and practices of French dentists toward antibiotic use in periodontal treatment. As no information related to the health of respondents was collected, this study was exempt from requiring ethical approval according to the current French legislation.

### 4.2. Study Population

Two profiles of dental practitioners have been defined: (i) first, those with a “specialized/orientated practice” (SOP) toward periodontology were recruited among members of the French Society of Periodontology and members of the College of University Teachers of Periodontology (CNEP) who have a part or full-time hospital/university practice. This sample included both dentists practicing periodontology and those merely showing a particular interest in periodontology. It is worth mentioning that periodontology is considered in France as an “oriented practice” and not as a recognized “specialty.” Therefore, a more precise identification of the periodontists was not possible since there is no list of practitioners exercising these activities on an exclusive or dominant basis. (ii) Second, the group of general dentists (GDP) included dentists from the French private dental practice-based research network (ReCOL).

These two groups were convenient samples chosen because of their anticipated differences (type of practice, access to postgraduate education, interest in periodontology) to help explore factors associated with antibiotic use in the practice environment. Previous studies suggest differences in antibiotic use between general dentists and dental specialists [47,49]. We were also interested in whether there is a difference between hospital and private practitioners.

### 4.3. Development of the Questionnaire

An anonymous self-administered questionnaire was developed from similar questionnaires found in the literature [47,48] (File available as Supplementary data). It consists of 21 questions divided into three parts. Questions are closed, single, or multiple choice, or in 4-point Likert scale type format. The first part explores the general and socio-demographic data, educational background, and type of activity. The second part contains questions related to systemic antibiotics. For each periodontal condition, the respondent was asked about (i) the indication to prescribe antibiotics as first- or second-line therapy; (ii) the frequency of use of antibiotics; (iii) the choice of antibiotic molecules using double entry tables. The third explores the use of local antibiotics through questions about (ii) the indication to prescribe local antibiotics as first- or second-line therapy; (iii) the frequency of use of local antibiotics; (iv) the motivations or barriers to the use of local antibiotics. In addition, self-reported knowledge of the most recent French national guidelines concerning the use of antibiotics in dentistry [57] and the history of specific training in the proper use of antibiotics is questioned.

The classification of periodontal diseases has recently been revised [80] but this survey uses the previous classification of periodontal diseases [81] to which the French national guidelines concerning the use of antibiotics in dentistry refer [57] and which remains the most popular among general dentists. The “New” disease classification framework is built upon notable changes from previous classifications. In particular, formerly “aggressive” and “chronic” periodontitis are now grouped under a single category with a distinction of stages, based on the severity and complexity of treatment and grades based on evidence of risk of rapid progression and anticipated treatment response.

### 4.4. Sample Size Calculation

The minimum sample size was determined based on the null hypothesis of the lowest expected value of r-squared R² in the multivariate analysis (i.e., the “worst-case” condition in which the minimum sample size is highest) using a power and sample size software [82] (pwr package [ver 1.3-0] R software, The R Foundation for statistical computing, Vienna, Austria). Power calculations for 80% power, null-hypothesis of R^2^ value of 0.1 and a final model containing five parameters yielded a required minimal sample size of 122 respondents.

### 4.5. Questionnaire Distribution

The questionnaire was first pre-tested among volunteer dentists and dental students to ensure consistency and modified based on their feedback. The purpose of the questionnaire was clearly described in the introduction to the survey. A more detailed document with, in addition to the objectives and hypotheses of the study, the expected results and perspectives was sent to the boards of SFPIO, CNEP, and ReCOL to obtain their approval of the project. First, questionnaires were handed out by two investigators (KA, TM) to the participants in the annual professional congress organized by the National College of Teachers in Periodontology of France 2019 in Lille, France. Then, the self-administered questionnaire was made available on the LimeSurvey software hosted on the website of the University of Lille from October 2020 and an invitation to participate in the survey was widely distributed using the newsletter and websites of SFPIO and ReCOL, the mailing list of SFPIO (3 recalls) and dental networks on Facebook. Two reminders were issued on dental social networks to increase the number of responses. Participants were instructed to complete the survey only once even if they received it through different media. Responses were recorded over a period of 3 months from the start of the online survey.

### 4.6. Statistical Analysis

The analyses were performed using the R software version 3.6.3. (The R Foundation for statistical computing, Vienna, Austria) For qualitative data, frequencies were expressed as percentages and statistical subgroup analysis was performed using chi-square tests for frequency comparison or Fisher exact tests when the conditions for applying chi-square were not met.

Multiple linear regression models were used to determine the factors associated with dentists’ knowledge or practice. First of all, bivariate statistical tests were performed using a significance threshold of 20%, in order to avoid excluding any potentially relevant factors to be included in the multivariate analysis. Two multiple linear regression models were then fitted. For this purpose, answers to the knowledge and practice sections were treated dichotomously and the variables “knowledge” and “practice” were defined by scores calculated as follows: (i) For knowledge: A score of 1 was assigned when the participant stated being aware of current national antibiotic prescribing recommendations or having received recent (<5 years) training in prescribing and a score of 0 otherwise (ii) A score of 1 was assigned for each question to which the dentist’s response (antibiotic use or not, choice of molecule) is in accordance with the national recommendations and a score of 0 was assigned if this was not the case. Thereafter, total knowledge and practice scores were calculated. All factors associated with the score to be explained at the threshold of 20% were incorporated in the first step of the development of the model. When there was collinearity between 2 factors significantly associated with the score, only one of them was incorporated. Stepwise method based on AIC (Akaike information criterion) was used to build the models. A threshold of 5% was used for interpretation of the fitted multivariate models.

## 5. Conclusions

This study shows the major trends in antibiotic use for periodontal therapy among French dentists and some discrepancies in prescribing patterns between general dentists and those with a specialized/orientated practice. It also highlights postgraduate education, hospital/academic practice, and the share of periodontics in the practice as key factors which improve knowledge and practices toward a more adequate use of antibiotics. Hopefully this will contribute to the updating of clear recommendations to guide dentists in choosing the most appropriate therapy and could inform the development and implementation of targeted antibiotic stewardship efforts in dentistry.

## Figures and Tables

**Table 1:** Characteristics of respondents.

Characteristics		*n*	%
Date of graduation (DDS)	<5 yrs	77	28.3
	5–10 yrs	57	21
	10–20 yrs	66	24.3
	>20 yrs	72	26.4
Location of graduation	France	241	88.6
	Abroad	31	11.4
Postgraduate background	Postgraduate certificate in periodontology	109	40.1
	Other university degree in periodontology and oral implantology	96	35.3
	Current or former clinical lectureship	75	25.6
	Attendance at specialty congresses (SFPIO/EFP)	183	67.3
	Other training	68	25
Type of professional practice	Academic position/ hospital activity	81	29.8
	Self-employed	224	82.3
	Salaried	75	25.6
	Other	10	3.7
“specialized/orientated” practice in Periodontology	Yes	155	57
	No	117	43
Type of periodontal care provided	Prophylaxis	263	96.7
	Non-surgical periodontal treatment	263	96.7
	Surgical periodontal treatment	177	65
Number of periodontitis patients treated per week	<5 patients per week	95	34.9
	5–10 patients per week	78	28.7
	>10 patients per week	99	36.4

Notes: yrs: years; *n*: Number of respondents; EFP: European Federation of Periodontology; Specialized/orientated practice includes means that the practitioner is affiliated to the SFPIO (French society of periodontology and oral implantology) and CNEP (French college of teachers in periodontology); General practice means that the practitioner did not declare membership in the SFPIO or CNEP. This include members of ReCOL (French dental network for clinical research in private practice or no affiliation) and practitioners with no affiliation disclosed.

**Table 2:** Use of local antibiotics delivery systems by French dentists.

Items		All Respondents (*n* = 272)		“Specialized/ Orientated” Practice (*n* = 155)		General Practice (*n* = 117)		
		*n*	%	*n*	%	*n*	%	*p*-Value
Knowledge of national guidelines		210	77.2	90	58.1	47	40.2	<0.01
Recent exposure to training (<5 yrs)		116	42.7	40	25.8	26	22.2	n.s.
Knowledge of the local antimicrobial products available on the market	Minocyclin (Parocline^®^)	188	69.1	114	73.5	74	63.2	n.s.
	Chlorhexidine (Periochip^®^)	83	30.5	63	40.6	20	17.1	<0.001
	Chlorhexidine + xantham (Chlo-Site^®^)	26	9.6	17	11	9	7.7	n.s.
	Neither	70	25.8	32	20.6	38	32.5	<0.05
Use of local antimicrobial systems	Minocyclin (Parocline^®^)	60	22.1	42	27.1	18	15.4	<0.05
	Chlorhexidine (Periochip^®^)	12	4.4	5	3.2	7	6	n.s.
	Chlorhexidine + xantham (Chlo-Site^®^)	2	0.7	1	0.6	1	0.9	n.s.
	Neither	203	74.6	109	70.3	94	80.3	n.s.
As first-line treatment	never	233	85.7	132	85.2	101	86.3	n.s.
	occasionally	32	11.8	19	12.2	13	11.1	
	often	4	1.5	2	1.3	2	1.7	
	very often	3	1.1	2	1.3	1	0.9	
As second-line treatment	never	204	75	108	69.7	96	82	n.s.
	occasionally	61	22.4	42	27.1	19	16.2	
	often	5	1.8	4	2.6	1	0.9	
	very often	2	0.7	1	0.6	1	0.9	
Barriers to application of local antimicrobials	Lack of EBD	133	49.4	77	49.7	56	47.9	n.s.
	Lack of experience	110	40.9	47	30.3	63	53.8	<0.001
	High cost	43	16	25	16.1	18	15.4	n.s.
	impractical	9	3.3	7	4.5	2	1.7	n.s.
	Lack of outcomes	52	19.3	37	23.9	15	12.8	<0.05
	Other	0	0	0	0	0	0	n.a.

Notes: Specialized/orientated practice means that the practitioner is affiliated to the SFPIO (French society of periodontology and oral implantology) and/or the CNEP (French college of University teachers in periodontology); General dental practice means that the practitioner did not declare membership in the SFPIO or CNEP. This includes members of ReCOL (French general dental practice-based research network) and practitioners with no affiliation disclosed; *p*-value: difference between specialized/orientated practice and general practice (As first line and as second line treatment: never/occasionally versus often/very often); n.s.: non-significant; n.a.: non-applicable.

**Table 3:** Use of local antibiotics in periodontal conditions.

Items		All Respondents		“Specialized/Orientated” Practice (*n* = 59)		General Practice (*n* = 38)		
		*n*	%	*n*	%	*n*	%	*p*-Value
Mild to moderate Periodontitis	never	87	91.6	52	88.1	35	92.1	n.s.
	occasionally	8	8.4	7	11.9	3	7.9	
	often	0	0	0	0	0	0	
	very often	0	0	0	0	0	0	
Severe Periodontitis	never	63	66.3	35	59.3	30	79	n.s.
	occasionally	28	29.5	21	35.6	7	18.4	
	often	1	1	0	0	1	2.6	
	very often	3	3.2	3	5.1	0	0	
Localized aggressive periodontitis	never	45	46.4	23	39	22	57.9	n.s.
	occasionally	37	38.1	25	42.4	12	31.5	
	often	10	10.3	8	13.5	2	5.3	
	very often	5	5.1	3	5.1	2	5.3	
Generalized aggressive periodontitis	never	59	62.8	32	54.2	30	78.9	n.s.
	occasionally	24	25.5	19	32.2	5	13.2	
	often	7	7.4	4	6.8	3	7.9	
	very often	4	4.2	4	6.8	0	0	
Necrotizing periodontitis	never	82	88.2	50	84.7	36	94.8	n.s.
	occasionally	6	6.4	5	8.5	1	2.6	
	often	3	3.2	2	3.4	1	2.6	
	very often	2	2.1	2	3.4	0	0	
Periodontal abscess	never	71	73.2	39	66.1	32	84.2	n.s.
	occasionally	19	19.6	14	23.7	5	13.2	
	often	5	5.1	4	6.8	1	2.6	
	very often	2	2.1	2	3.4	0	0	

Notes: Specialized/orientated practice means that the practitioner is affiliated to the SFPIO (French society of periodontology and oral implantology) and CNEP (French college of teachers in periodontology); General practice means that the practitioner did not declare membership in the SFPIO or CNEP. This includes members of ReCOL (French dental network for clinical research in private practice or no affiliation) and practitioners with no affiliation disclosed; *p*-value: difference between specialized/orientated practice and general practice: never/occasionally versus often/very often); n.s.: non-significant.

**Table 4:** Factors associated with higher knowledge score about antibiotics.

	Unstandardized Coefficients		Standardized Coefficients	*t*	*p*-Value
	B	Std. Error	*beta*		
Number of periodontitis patients treated per week	0.381	0.263	0.224	9.715	<0.001
Date of graduation (DDS)	−0.212	0.071	−0.171	3.625	0.003
Post graduate certificate in periodontology	0.423	0.185	0.144	2.290	0.023
Specialized/orientated practice	0.287	0.180	0.099	1.592	0.112

Notes: Coefficients are relative to the intercept: 2.556 ± 0.263, *t* = 9.715, *p* < 0.001. *n* =272 values, R^2^ = 0.134, *p* < 0.001, B = unstandardized regression coefficient beta, *t* = *t*-test, a *p*-value < 0.05 was considered as significant. Factors tested but not included in the final model: other university degree in Periodontology and Implantology, other type of professional practice.

**Table 5:** Factors associated with higher practice scores.

	Unstandardized Coefficients		Standardized Coefficients	*t*	*p*-Value
	B	Std. Error	*beta*		
Date of graduation (DDS)	−0.408	0.108	−0.208	−3.775	<0.001
Post graduate certificate in periodontology	0.695	0.286	0.149	2.428	0.016
Academic position/ hospital activity	1.298	0.290	0.277	4.477	<0.001

Notes: Coefficients are relative to the intercept: 12.489 ± 0.324, *t* = 38.576, *p* < 0.001. *n* = 272 values, R^2^ = 0.179, *p* < 0.001, B= unstandardized regression coefficient beta, *t* = *t*-test, a *p*-value < 0.05 was considered as significant. Factors tested but not included in the final model: knowledge score, specialized/orientated practice, number of periodontitis patients treated per week, other university degree in Periodontology and Implantology, other type of professional practice.

## Data Availability

The data are not publicly available because in the protocol submitted to the Data Protection Officer of the University of Lille, France, the authors confirmed that only researchers involved in the survey will have access to the raw data.
